# Antileishmanial Phenylpropanoids from the Leaves of *Hyptis pectinata* (L.) Poit

**DOI:** 10.1155/2013/460613

**Published:** 2013-07-28

**Authors:** Rosangela A. Falcao, Patricia L. A. do Nascimento, Silvana A. de Souza, Telma M. G. da Silva, Aline C. de Queiroz, Carolina B. B. da Matta, Magna S. A. Moreira, Celso A. Camara, Tania M. S. Silva

**Affiliations:** ^1^Laboratório de Bioprospecção Fitoquímica, Departamento de Ciências Moleculares, Universidade Federal Rural de Pernambuco, 52171-900 Recife, Pernambuco, Brazil; ^2^Laboratório de Farmacologia e Imunidade, Instituto de Ciências Biológicas e da Saúde, Universidade Federal de Alagoas, 57072-970 Maceió, Alagoas, Brazil

## Abstract

*Hyptis pectinata*, popularly known in Brazil as “sambacaitá” or “canudinho,” is an aromatic shrub largely grown in the northeast of Brazil. The leaves and bark are used in an infusion for the treatment of throat and skin inflammations, bacterial infections, pain, and cancer. Analogues of rosmarinic acid and flavonoids were obtained from the leaves of *Hyptis pectinata* and consisted of two new compounds, sambacaitaric acid (**1**) and 3-*O*-methyl-sambacaitaric acid (**2**), and nine known compounds, rosmarinic acid (**3**), 3-*O*-methyl-rosmarinic acid (**4**), ethyl caffeate (**5**), nepetoidin A (**6**), nepetoidin B (**7**), cirsiliol (**8**), circimaritin (**9**), 7-*O*-methylluteolin (**10**), and genkwanin (**11**). The structures of these compounds were determined by spectroscopic methods. Compounds **1–5**, and **7** were evaluated *in vitro* against the promastigote form of *L. braziliensis*, and the ethanol extract. The hexane, ethyl acetate, and methanol-water fractions were also evaluated. The EtOH extract, the hexane extract, EtOAc, MeOH:H_2_O fractions; and compounds **1**, **2** and **4** exhibited antileishmanial activity, and compound **1** was as potent as pentamidine. In contrast, compounds **3**, **5**, and **7** did not present activity against the promastigote form of *L. braziliensis* below 100 *µ*M. To our knowledge, compounds **1** and **2** are being described for the first time.

## 1. Introduction

 The Lamiaceae family is cosmopolitan and comprises 236 genera and 7173 species [1]. This group is well known for its essential oils [2], which are rich in terpenoids, especially the subfamily Nepetoideae. In South America, *Hyptis* is one of the main genera of this subfamily and comprises 280 species. Of these species, 146 are endemic to Brazil [3].

*Hyptis pectinata* (L.) Poit, subfamily Nepetoideae, which is popularly known in Brazil as “sambacaitá” or “canudinho,” is a widespread, aromatic shrub that is largely grown in the northeast of Brazil [4]; it is an herbaceous plant with aromatic leaves and small bilabial flowers that are clustered into axillary inflorescences [5]. Although there are some reports on the constituents of *H. pectinata*, those studies [6] mainly focused on the essential oil composition [7]. *H. pectinata* is particularly used in folk medicine for various conditions, such as rhinopharyngitis, nasal congestion, certain skin diseases [8], gastric disorders, fever [9], and bacterial infections [10]. The leaves and bark are used in an infusion for the treatment of throat and skin inflammations, bacterial infections, pain and cancer [11–13]. 

 The healing effect of *H. pectinata* suggests that this plant may have antileishmanial action. Leishmaniasis is a major global public health problem, with three million cases annually [14]. American tegumentary leishmaniasis (ATL) is a serious zoonosis and is endemic throughout considerable areas of Latin America [15]. The main clinical forms of ATL are cutaneous leishmaniasis, mucosal or mucocutaneous leishmaniasis, and diffuse cutaneous leishmaniasis. In Brazil, ATL is found in all states and has shown a high incidence over the last 20 years; furthermore, the genetic diversity among *Leishmania* parasites is great. At least seven Brazilian *Leishmania* species have been described as the etiological agent of human cutaneous disease, with most cases being caused by *Leishmania* (Viannia) *braziliensis* [16–18].

 The drugs that are commercially used for the treatment of Leishmaniasis are highly toxic and require hospital monitoring because they may lead to death [19]. In this context, research on natural products for the treatment of leishmaniasis has been encouraged by the (World Health Organization) WHO through the Tropical Diseases Program [20].

**Figure 1:**
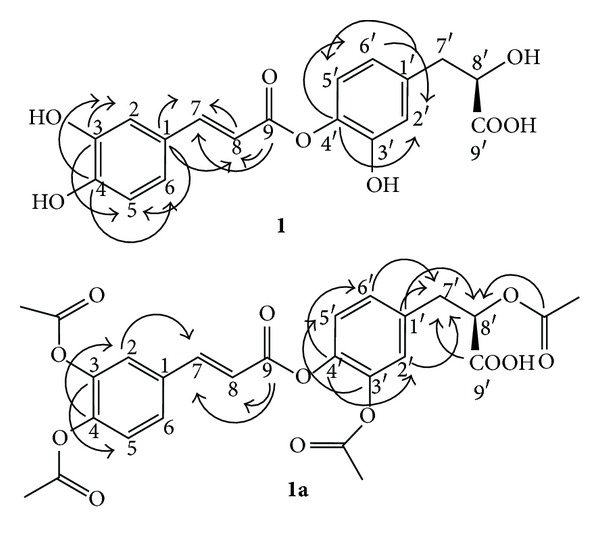
Key HMBC correlations of compounds **1** and **1a**.

**Figure 2:**
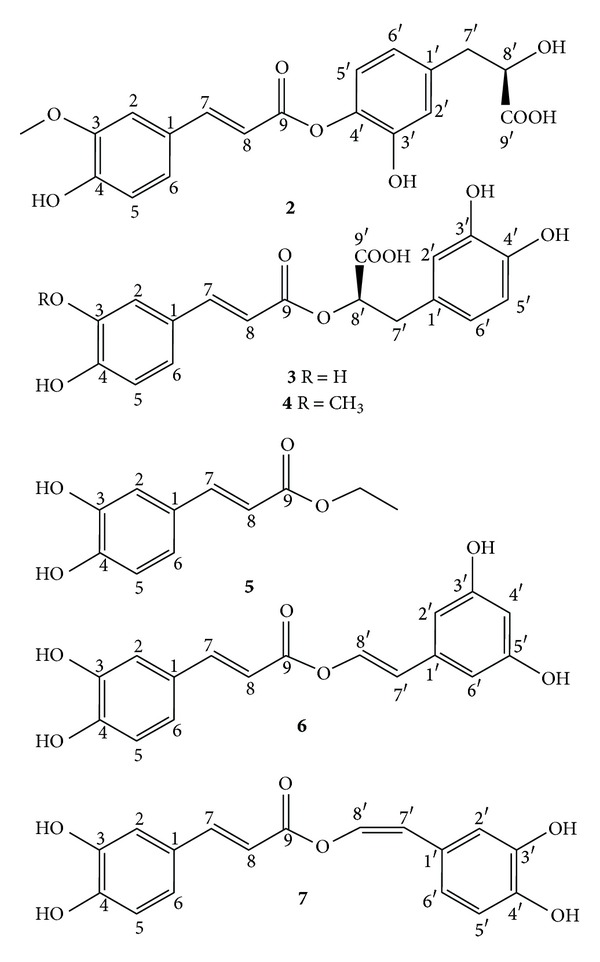
Chemical structures of compounds (**2**–**7**) isolated from *H. pectinata*.

**Figure 3:**
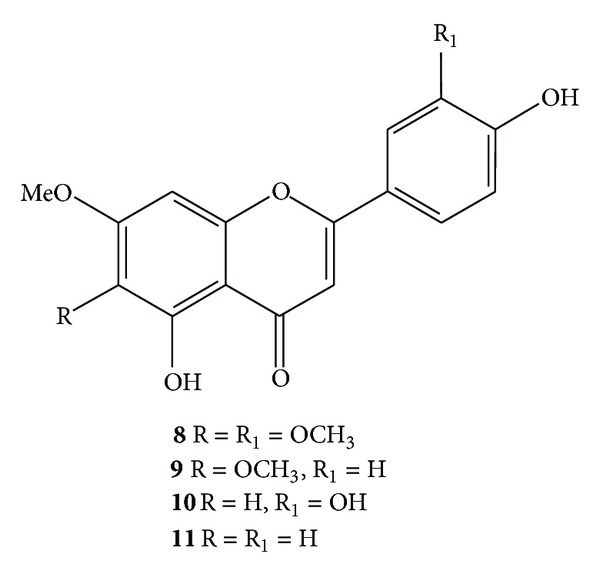
Chemical structures of compounds (**8**–**11**) isolated from *H. pectinata*.

 The current work led to the isolation of two new compounds, namely sambacaitaric acid (**1**) and 3-*O*-methyl-sambacaitaric acid (**2**) (Figure 1), and nine known compounds from *H. pectinata*. **1**–**7** were phenylpropanoids, and **8**–**11** were flavonoids (Figures 2 and 3). The EtOH extract; the hexane, EtOAc, and MeOH:H_2_O fractions; and compounds **1**–**5** and **7** were evaluated *in vitro* against the promastigote form of *L. braziliensis*.

## 2. Materials and Methods

### 2.1. General

The infrared absorption spectra were recorded in KBr pellets using a Varian 640 FT-IR spectrophotometer with a PIKE ATR accessory operating in the 4000–400 cm^−1^ range. The LC-ESI-MS was performed in negative electrospray mode using an Esquire 3000 Plus (Bruker), and the HRESIMS was conducted using a MicroTOF (Bruker). Silica gel 60 F_254_ (Merck) for TLC plates. Sephadex LH-20 (Sigma) was employed for gel permeation chromatography. ^1^H and ^13^C NMR spectra were obtained using a Bruker DRX 500 (500 MHz for ^1^H and 125 MHz for ^13^C) and Bruker DPX300 (300 MHz for ^1^H and 75 MHz for ^13^C) in DMSO-*d*_6_. The CD was recorded with a Jasco J-515 CD spectrometer. The optic rotation was determined in a KRUESS OPTRONIC spectrometer. All solvents used are of commercial HPLC grade.

### 2.2. Plant Material

The leaves of *Hyptis pectinata *were collected in Garanhuns city, State of Pernambuco, Brazil, from April to July 2010. A voucher specimen is deposited at the Instituto de Pesquisa Agropecuária (IPA), Pernambuco, Brazil.

### 2.3. Extraction and Isolation

The plant material was successively extracted with EtOH to obtain 7.0 g of dry extract. This extract was dissolved in MeOH : H_2_O (1 : 1) and successively fractionated with hexane and EtOAc. A portion of the EtOAc fraction (3.5 g) was subjected to chromatography on a Sephadex LH-20 column with methanol as the mobile phase. Compounds **1** (44.2 mg), **2** (54.0 mg), **3** (97.9 mg), **4** (26.0 mg), **5** (24.9 mg), **6** (20.0 mg), **7** (28.3 mg), **8** (22.4 mg), **9** (14.0 mg), **10** (11.0 mg), and **11** (7.4 mg) were then purified by semipreparative HPLC on a Luna Phenomenex RP-18 column (21 mm × 250 mm × 5 *μ*m) and detected at 320 nm at a flow rate of 16 mL/min using a mobile phase of H_2_O (A) and methanol (B) with the following pattern: from 0–10 min, 40–60% B; to 25 min, 80% B; and to 28 min, 100% B. The purity of the compounds was examined via analytical HPLC with diode array detection.

### 2.4. *In Vitro* Activity against *Leishmania braziliensis*

Promastigotes of *L. braziliensis* (MHOM/BR/87/BA125) were obtained from Dr. Valéria de Matos Borges at the Gonçalo Moniz Research Center. The parasites were maintained *in vitro* in Schneider's medium supplemented with 10% FBS and 2% human urine. Stock solutions of the EtOH extract; the hexane, EtOAc, and MeOH:H_2_O fractions; and compounds **1**–**5** and **7** from *H. pectinata*, as well as pentamidine (the reference leishmanicidal drug), were prepared in DMSO immediately before use. The cytotoxicities of the extract, fractions, and compounds against the promastigotes were determined. Stationary phase *L. braziliensis* promastigotes were plated in 96-well vessels (Nunc) at 1 × 10^5^ cells per well in Schneider's medium supplemented with 10% FBS and 2% human urine. Each compound solution was added at increasing concentrations (0.001–100 *μ*g/mL for the extract and fractions; 0.001–100 *μ*M for the compounds). Cells were also cultured in a medium without compounds and vehicle (basal growth control) or with DMSO 0.1% (vehicle control). After 48 h, the extracellular load of *L. braziliensis* promastigotes was estimated by counting the promastigotes in Schneider's medium with a CELM automatic cell counter (model CC530) [21].

## 3. Results and Discussion

Upon extraction and fractionation, the leaves of *Hyptis pectinata *yielded compounds **1**–**11** (Figures 1–3). Compounds **1** and **2** were identified as new compounds and as rosmarinic acid analogues, based on the detailed NMR analysis described below (Table 1). Compound **1** was obtained as a yellowish, amorphous powder, and its optical rotation was [*α*]_*D*_ = +30 (*c* 0.001, MeOH). Its molecular formula was deduced to be C_18_H_16_O_8_ by HRESIMS, which showed a molecular ion peak [M-H]^+^ at *m/z *359.0759 (Calcd *m/z *for C_18_H_15_O_8_, 359.0761). The UV spectrum exhibited signals at 322, 296, and 239 nm, and the IR spectrum showed signals at 3435, 1628, 1524, and 1405 cm^−1^. The ^1^H and ^13^C NMR spectra of **1** were similar to those of rosmarinic acid. 

**Figure 4:**
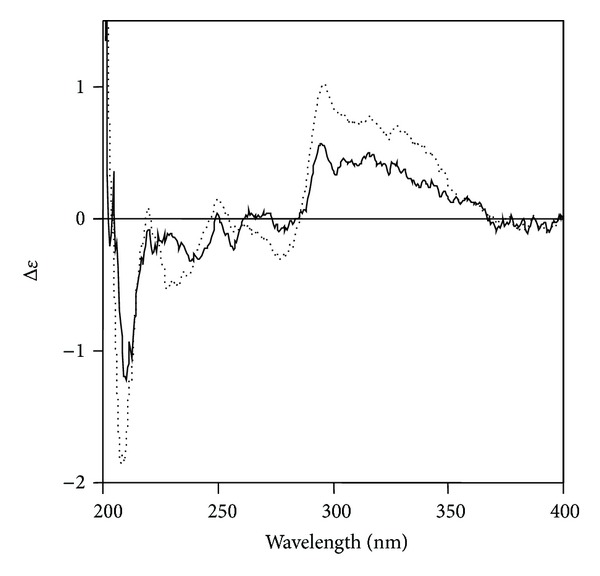
CD spectra of **1 **(dotted line) and **3** (solid line).

 In the ^1^H-NMR spectrum of **1**, two sets of ABX proton signals at (**δ** 7.03, d, *J* = 2.0 Hz; 6.74, d, *J* = 8.5 Hz; 6.92, dd, *J* = 8.5, 2.0 Hz) and (**δ** 6.66, d, *J* = 2.0 Hz; 6.59, d, *J* = 8.5 Hz; 6.48, dd, *J* = 8.5, 2.0 Hz) and two olefinic proton signals at **δ** 7.34 (d, *J* = 17.0 Hz) and 6.18 (d, *J* = 17.0 Hz) were observed. In the aliphatic region, there were three proton signals at **δ** 4.85 (m), 3.01 (m), and 2.74 (m). The ^13^C-NMR spectrum showed 18 carbon signals. In the heteronuclear multiple bond correlation (HMBC) spectrum, the H-7 proton signal (**δ** 7.34) was long range coupled with aromatic carbons at **δ** 125.58 (C-1), 114.23 (C-2), and 119.4 (C-6), an olefinic carbon at **δ** 114.90 (C-8) and the carbonyl carbon at **δ** 166.22 (C-9) (Figure 1). The absolute configuration of **1** was determined by CD spectroscopy. Because the chiral center and its immediate environment are identical to those of rosmarinic acid **3** (Figure 4), one would expect a similar CD spectrum if the configuration around C-8′ in **3** was the same as in **1**. On the basis of these observations, the structure of compound (**1**) was established to be isoferuloyl-4′-(3′-hydroxyphenyl)-(8′*R*)-lactic acid, and the compound was named sambacaitaric acid. 

 The sambacaitaric acid (**1**) was treated with Ac_2_O/pyridine to yield the peracetyl derivative **(1a)**. The ^1^H and ^13^C NMR spectral data of **1a**, obtained through the analysis of extensive 1D and 2D NMR experiments (Figure 1 and Table 1), was also used to confirm the postulated structure of **1**. Electrospray ionization mass spectroscopy (ESI-MS) of **1a** showed the [M-H]^+^ at *m/z *527, corresponding to the molecular formula C_26_H_24_O_12_.

 Compound **2** was obtained as a yellowish, amorphous powder and showed positive optical rotation, [*α*]_*D*_ = +10 (*c* 0.1, MeOH). Its molecular formula was deduced to be C_19_H_18_O_8_, which produced the [M-H]^+^ peak at *m/z* 373. The UV spectrum exhibited signal at 340 nm and the IR spectrum showed signals at 3420, 1680, and 1607 cm^−1^. The ^1^H and ^13^C NMR spectra were similar to those of **1** with the addition of a methoxyl group on the **3** position. Thus, the structure of this new compound was established as 3-*O*-methyl-sambacaitaric acid. 

 The known compounds were identified from the spectroscopic data (UV, IR, ESIMS, and NMR) to be rosmarinic acid (**3**), 3-*O*-methyl-rosmarinic acid (**4**) [22], ethyl caffeate (**5**) [23], nepetoidin A (**6**), nepetoidin B (**7**) [24], cirsiliol (**8**), [25] circimaritin (**9**) [26], 7-*O*-methylluteolin (**10**) [27], and genkwanin (**11**) [28].

 Regarding compounds **6** and **7** (nepetoidins A and B, resp.), according to Grayer et al. [29], the presence of this pair of caffeic acid esters is chemotaxonomically significant for distinguishing the Nepetoideae from the other subfamilies of Lamiaceae and related families.

 This is the first occurrence of flavonoid **8** (cirsiliol) in the *Hyptis* genus; however, it has also been found in the Labiateae family in the *Sideritis*, *Stachys*, *Teucrium* and *Rosmarinus* genera. According to Tomás-Barberán and wollenweber [30], these compounds are externally located and are dissolved in a terpenoid matrix, and they have been found in larger amounts in species that grow in xeric habitats. Flavonoids **9** (circimaritin) and **11** (genkwanin) were isolated from *Hyptis fasciculata*, a native species of Brazil, Argentina, and Uruguay [31]. **10** (7-*O-*methylluteolin) was reported for the first time in the genus *Hyptis*.

 To evaluate and compare the leishmanicidal profile of *H. pectinata*, the EtOH extract; the hexane, EtOAc, and MeOH:H_2_O fractions; and the compounds isolated in major quantities (**1**–**5** and **7**) were evaluated *in vitro* against the promastigote form of *L. braziliensis*. The maximum effect and the IC_50_ value (the concentration of sample causing 50% reduction in survival/viability of the parasites) were used as the parameters for antileishmanial activity (Table 2).

 The EtOH extract; the hexane, EtOAc, and MeOH:H_2_O fractions; and compounds **1**, **2,** and **4** exhibited antileishmanial activity, with maximum effects of 91.6 ± 2.5, 90.0 ± 3.6, 81.5 ± 5.9, 61.5 ± 1.2, 56.0 ± 0.8, 48.8 ± 1.7, and 69.1 ± 2.7%, respectively. Moreover, the EtOH extract (IC_50_ = 0.7 ± 0.1 *μ*g/mL), the EtOAc fraction (IC_50_ = 0.4 ± 0.1 *μ*g/mL), the hexane fraction (IC_50_ = 0.2 ± 0.1 *μ*g/mL), compound **1** (IC_50_ = 6.9 ± 0.7 *μ*M/2.5 ± 0.04 *μ*g/mL), and compound **4** (IC_50_ = 5.4 ± 0.8 *μ*M/2.0 ± 0.3 *μ*g/mL) were as potent as pentamidine (which has an efficacy of 93.5 ± 0.7% and IC_50_ = 0.9 ± 0.03 *μ*M/0.3 ± 0.01 *μ*g/mL). In contrast, compounds **3**, **5**, and **7** did not present activity against the promastigote form of *L. braziliensis* below 100 *μ*M.

 Several polyphenols with promising antileishmanial effects have been reported [32, 33]. Concerning the structure-activity relationship, it would appear that methoxylation of sambacaitaric acid on C_3_ diminished the efficacy and potency. However, the absence of the methoxyl group on the **3** position in rosmarinic acid (**3**) abolished the leishmanicidal activity against the promastigote form of *L. braziliensis*. Moreover, Radtke [34] demonstrated that rosmarinic acid did not show selective toxicity when tested against the promastigote stages of the other Leishmania species (*L. major, L. donovani, L. guyanensis*, and *L. killicki*) but did exhibit moderate antileishmanial activity against intracellular amastigotes. Although the caffeic acid esters assessed in this study (**5** and **7**) have not shown leishmanicidal activity against the promastigotes of *L. braziliensis* [35] they showed that other caffeic acid esters (1-methylbutyl caffeate, 1′-methylhexyl caffeate and 1′-methyloctyl caffeate) were active against the axenic amastigote forms of *L. amazonensis*, with IC_50_ values of 2.0 ± 0.1, 10.0 ± 0.4 and 1.8 ± 0.1 *μ*M, respectively.

Sambacaitaric acid (**1**), [*α*]_*D*_ = +30 (*c* 0.001, MeOH), IR (KBr) *ν*_max⁡_: 3435 (OH), 1658 (C=O), 1524, (C=C from aromatic rings). ^1^H NMR (DMSO-*d*_6_, 300 MHz, see Table 1), ^13^C NMR (DMSO-*d*_6_, 75 MHz, see Table 1). HRESIMS (negative mode) *m/z *359.0761 [M-H]^+^ (C_18_H_15_O_8_).

3-O-methyl-sambacaitaric acid (**2**), [*α*]_*D*_ = +10 (c 0.001, MeOH), ^1^H NMR (300 Mz, DMSO): *δ* 7.35 (1H, d, *J* = 15.9 Hz, H-7); 7.01 (1H, d, *J* = 2.1 Hz, H-2), 6.91 (1H, dd, *J* = 8.1; 2.1 Hz, H-6), 6.73 (1H, d, *J* = 8.1 Hz, H-5), 6.66 (1H, d, *J* = 2.1 Hz, H-2′), 6.58 (1H, d, *J* = 8.1 Hz, H-5′), 6.48 (1H, dd, *J* = 8.1; 2.1 Hz, H-6′), 6.18 (1H, d, *J* = 15.9 Hz, H-8), 8.83 (1H, m; H-8′), 3.02 and 2.73 (2H, m, H-7′). ^13^C NMR (75 Mz, DMSO): *δ* 172.6 (C-9′), 166.7 (C-9), 148.2 (C-4), 146.3 (C-3), 145.3 (C-3′), 144.5 (C-7), 143.89 (C-4′), 130.5 (C-1′), 126.1 (C-1), 121.3 (C-6), 120.1 (C-6′), 116.9 (C-2′), 116.3 (C-5), 115.8 (5′), 115.5 (C-2), 115.3 (C-8), 76.6 (C-8′), 56.9 (OCH3), 37.8 (C-7′). 

Rosmarinic acid (**3**), [*α*]_*D*_ = +10 (*c* 0.001, MeOH), UV *λ*_max⁡_ 242, 324. IR (KBr) *v*_max⁡_: 3382 (OH), 1697 (C=O), 1606, 1522 (C=C from aromatic rings). LC-ESI-MS (negative mode) *m/z *359 [M-H]^+^ (C_18_H_16_O_8_).

3-*O*-methyl-rosmarinic acid (**4**), [*α*]_*D*_ = +10 (*c* 0.001, MeOH), UV *λ*_max⁡_ 253, 340. IR (KBr) *v*_max⁡_: 3394 (OH), 1692 (C=O), 1603, 1520 (C=C from aromatic rings). LC-ESI-MS (negative mode) *m/z *373 [M-H]^+^ (C_19_H_18_O_8_).

Ethyl caffeate (**5**) UV *λ*_max⁡_ 283, 337. IR (KBr) *v*_max⁡_: 3397 (OH), 1678 (C=O), 1605, 1520 (C=C from aromatic rings). LC-ESI-MS (negative mode) *m/z *207 [M-H]^+^ (C_11_H_12_O_4_).

Nepetoidin A (**6**) UV *λ*_max⁡_ 249, 340. IR (KBr) *v*_max⁡_: 3418 (OH), 1680 (C=O), 1603, 1520 (C=C from aromatic rings). LC-ESI-MS (negative mode) *m/z* 313 [M-H]^+^ (C_17_H_14_O_6_).

Nepetoidin B (**7**) UV *λ*_max⁡_ 251, 340. IR (KBr) *v*_max⁡_: 3382 (OH), 1701 (C=O), 1604, 1516 (C=C from aromatic rings). LC-ESI-MS (negative mode) *m/z* 313 [M-H]^+^ (C_17_H_14_O_6_).

Cirsiliol (**8**) UV *λ*_max⁡_ 272, 346. IR (KBr) *v*_max⁡_: 3419 (OH), 1650 (C=O), 1600, (C=C from aromatic rings). LC-ESI-MS (negative mode) *m/z* 329 [M-H]^+^ (C_17_H_14_O_7_).

Circimaritin (**9**) UV *λ*_max⁡_ 274, 336. IR (KBr) *v*_max⁡_: 3434 (OH), 1652 (C=O), 1599, (C=C from aromatic rings). LC-ESI-MS (negative mode) *m/z* 313 [M-H]^+^ (C_17_H_14_O_6_).

7-O-methylluteolin (**10**) UV *λ*_max⁡_ 254, 349. IR (KBr) *v*_max⁡_: 3397 (OH), 1664 (C=O), 1601, 1507 (C=C from aromatic rings). LC-ESI-MS (negative mode) *m/z *299 [M-H]^+^ (C_16_H_12_O_6_).

Genkwanin (**11**) UV *λ*_max⁡_ 267, 338. IR (KBr) *v*_max⁡_: 3445 (OH), 1670(C=O), 1608, 1504 (C=C from aromatic rings). LC-ESI-MS (negative mode) *m/z *283 [M-H]^+^ (C_16_H_12_O_5_).

## 4. Conclusions 

The chemical study of leaves from *Hyptis pectinata* resulted in the isolation of two new compounds, sambacaitaric acid (**1**) and 3-*O*-methyl-sambacaitaric acid (**2**), and nine known compounds, rosmarinic acid (**3**), 3-*O*-methyl-rosmarinic acid (**4**), ethyl caffeate (**5**), nepetoidin A (**6**), nepetoidin B (**7**), cirsiliol (**8**), circimaritin (**9**), 7-*O*-methylluteolin (**10**), and genkwanin (**11**). The EtOH extract, the hexane, EtOAc, and MeOH:H_2_O fractions; and compounds **1**, **2,** and **4** exhibited antileishmanial activity; compound **1** was as potent as pentamidine. In contrast, compounds **3**, **5**, and **7** did not present activity against the promastigote form of *L. braziliensis* below 100 *μ*M. The activity of the EtOAc fraction can be partially attributed to the isolated compounds **1**, **2,** and **4**.

## Figures and Tables

**Table 1:** ^
1^H (300 MHz) and ^13^C NMR (75 MHz) spectroscopic data for **1 **and **1a** (DMSO-*d*_6_, *δ* in ppm).

	**1**				**1a**			
Position	*δ*_C_	δ_H_	^2^J_CH_	^3^J_CH_	δ_C_	*δ*_ H_	^2^J_CH_	^3^ *J*_CH_
9′	172.1				172.5		H-8′	H-7′
9	166.2		H-8	H-7	166.3		H-8	H-7
4	148.4		H-5	H-2, H-6	149.4			H-2
3	145.81		H-2	H-5	138.53		H-2	H-5
4′	144.9		H-5′	H-2′, H-6′	141.8			H-2′, H-6′
3′	143.5				141.0		H-2′	H-5′
1′	129.9			H-5′	134.8		H-7′	H-8′
1	125.6		H-7	H-5, H-8	127.4		H-2, H-6, H-7	H-5, H-8
7	144.34	7.34 (d, 16.0)	H-6	H-2, H-8	145.3	7.56 (d, 16.0)		
6	120.8	6.92 (dd, 8.5; 2,0)		H-2, H-7	126.6	7.24 (sl)		
6′	119.7	6.48 (dd, 8.0; 2.0)			127.6	7.14 (sl)		H-7′
2′	116.6	6.66 (d, 2.0)		H-6′	124.2	7.16 (s)		H-2′, H-7′
5	115.9	6.74 (dd, 8.5; 2,0)			117.9	6.32 (d, 8.0)		
5′	115.4	6.59 (dd, 8.5; 2,0)			117.1	7.11 (m)		
2	114.9	7.03 (d, 2.0)			123.38	7.11 (m)		H-7
8	114.9	6.18 (d, 16.0)	H-7		115.3	6.29 (d, 16.0)		
8′	75.9	4.85 (m)			72.3	5.4 (m)	H-8′	
7′	37.2	3.01 (m), 2.74 (m)			36.6	3.24 (m)		
OCOCH_3_					168.3–169.1			
OCOCH_3_					20.7–20.9	2.26–2.34 (s)		

**Table 2:** Effect of extract, fractions, and compounds isolated from *H.  pectinata* against promastigotes of *L. braziliensis*.

Treatment	IC_50_^a^ (concentration ± S.E.M.)	Maximum effect (% ± S.E.M.)
Pentamidine	0.9 ± 0.03 *µ*M/0.3 ± 0.01 *µ*g/mL	93.5 ± 0.7**
EtOH extract	0.7 ± 0.1 *µ*g/mL	91.6 ± 2.5**
MeOH : H_2_O fraction	3.9 ± 1.5 *µ*g/mL	61.5 ± 1.2**
AcOEt fraction	0.4 ± 0.1 *µ*g/mL	81.5 ± 5.9**
Hexane fraction	0.2 ± 0.1 *µ*g/mL	90.0 ± 3.6**
**1**	6.9 ± 0.7 *µ*M/2.5 ± 0.04 *µ*g/mL	56.0 ± 0.8**
**2**	>100 *µ*M/>36.0 *µ*g/mL	48.8 ± 1.7**
**3**	>100 *µ*M/>36.0 *µ*g/mL	NA
**4**	5.4 ± 0.8 *µ*M/2.0 ± 0.3 *µ*g/mL	69.1 ± 2.7**
**5**	>100 *µ*M/>20.8 *µ*g/mL	NA
**7**	>100 *µ*M/>31.4 *µ*g/mL	NA

Data are reported as the mean ± S.E.M. Differences with a *P value < 0.05 were considered significant relative to the 0.1% DMSO group.

^a^IC_50_ is the concentration required to give 50% mortality, calculated by linear regression analysis from the *Kc* values at the concentrations employed.

NA: the compound is not active.
